# Synthesis and Antimicrobial Evaluation of Some Novel Thiazole, Pyridone, Pyrazole, Chromene, Hydrazone Derivatives Bearing a Biologically Active Sulfonamide Moiety

**DOI:** 10.3390/ijms15011237

**Published:** 2014-01-17

**Authors:** Elham S. Darwish, Azza M. Abdel Fattah, Fawzy A. Attaby, Oqba N. Al-Shayea

**Affiliations:** Department of Chemistry, Faculty of Science, Cairo University, Giza 12613, Egypt; E-Mails: azza682000@yahoo.com (A.M.A.F.); fattaby@hotmail.com (F.A.A.); org.chemo@yahoo.com (O.N.A.-S.)

**Keywords:** sulfamoyl, acrylamide, Pyrazole, pyridones, thiophene, thiazole, antimicrobial activity

## Abstract

This study aimed for the synthesis of new heterocyclic compounds incorporating sulfamoyl moiety suitable for use as antimicrobial agents via a versatile, readily accessible *N*-[4-(aminosulfonyl)phenyl]-2-cyanoacetamide (**3**). The 2-pyridone derivatives were obtained via reaction of cyanoacetamide with acetylacetone or arylidenes malononitrile. Cycloaddition reaction of cyanoacetamide with salicyaldehyde furnished chromene derivatives. Diazotization of **3** with the desired diazonium chloride gave the hydrazone derivatives **13a**–**e**. Also, the reactivity of the hydrazone towards hydrazine hydrate to give Pyrazole derivatives was studied. In addition, treatment of **3** with elemental sulfur and phenyl isothiocyanate or malononitrile furnished thiazole and thiophene derivatives respectively. Reaction of **3** with phenyl isothiocyanate and KOH in DMF afforded the intermediate salt **17** which reacted *in situ* with 3-(2-bromoacetyl)-2*H*-chromen-2-one and methyl iodide afforded the thiazole and ketene *N*,*S*-acetal derivatives respectively. Finally, reaction of **3** with carbon disulfide and 1,3-dibromopropane afforded the *N*-[4-(aminosulfonyl) phenyl]-2-cyano-2-(1,3-dithian-2-ylidene)acetamide product **22**. All newly synthesized compounds were elucidated by considering the data of both elemental and spectral analysis. The compounds were evaluated for both their *in vitro* antibacterial and antifungal activities and showed promising results.

## 1. Introduction

Cyanoacetamides and their related heterocyclic derivatives have generated a great deal of attention due to their interesting biological and therapeutic value; their pharmaceutical activities include: antimicrobial [1,2], antifungal [3], insulin releasing [4], carbonic anhydrase inhibitory [5], anti-inflammatory [6], and antitumor properties [7]. Some active sulfonamides as antibacterials are also known for their immunmodifying effects [8,9]. In addition, several thiazole derivatives possess important pharmacological activities and therefore they are useful materials in drug research. Over the past few decades, the literature has been enriched with progressive findings about the anticonvulsant activities of various substituted thiazole derivatives [10–14] that are of interest as potential neuroprotective agents [15,16]. Some 2-pyridones are also reported to possess antitumor [17], antibacterial [18] and other biological activities [19,20]. In view of these facts and as a continuation of our research program on the chemistry of butanamide [21,22], the present investigation aimed to synthesize and characterize newer hydrazones, pyridones, acrylamide, Pyrazole, thiadiazole, and thiophene incorporating sulfonamide moiety. It was found that *N*-[4-(aminosulfonyl)phenyl]-2-cyanoacetamide (**3**) is an excellent building block for the synthesis of the target objectives.

## 2. Results and Discussion

**Scheme 1.**
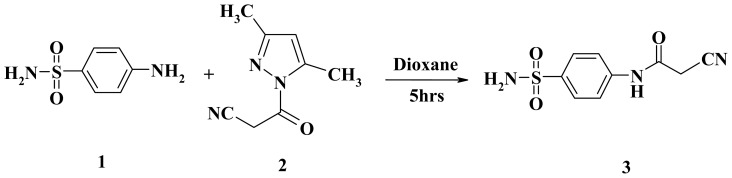
Synthesis of cyanoacetamide **3**.

One reason of our interest in amines is related to the conversion possibility of their NH_2_ group to the NHCOCH_2_CN group, which leads to cyanoacetamide with further useful functionalization at this position [23,24]. Cyanoacetamide **3** was synthesized by cyanoacetylation of **1** with 3,5-dimetyl-1-cyanoacetyl Pyrazole (**2**) [25] as previously described (Scheme 1).

**Scheme 2.**
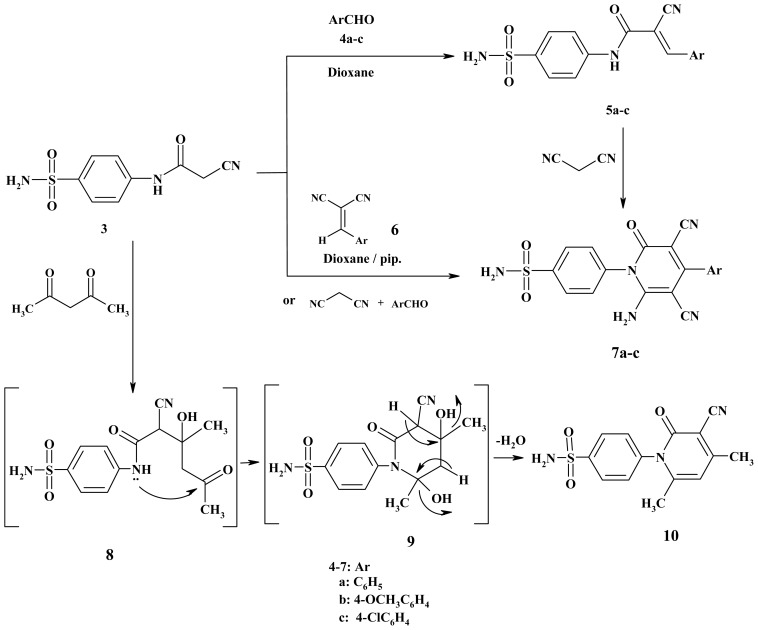
Synthesis of arylidene **5a**–**c** and pyridones **7a**–**c** and **10**.

Thus, the Knoevengel condensation of the cyanoacetamide **3** with aromatic aldehydes namely benzaldehyde, *p*-anisaldehyde, and *p*-chlorobenzaldehyde furnished the corresponding arylidene derivatives **5a**–**c** (Scheme 2). The IR spectrum of compound **5a**, taken as a typical example of the series prepared, revealed absorption bands at 1680, 2220 and 3362 cm^−1^ corresponding to carbonyl, nitrile and NH functions, respectively. Its ^1^H-NMR spectrum showed signals at δ 8.32 and 10.70 (D_2_O-exchangeable) due to CH and NH protons in addition to two aromatic protons at δ 7.81–7.83. Its mass spectrum showed a molecular ion peak at *m*/*z* 327. Pyridin-2(1*H*)-ones **7a**–**c** was obtained through the reaction of the arylidene derivatives **5a**–**c** with malononitrile in dioxane containing piperidine as catalyst. One-pot reactions of the cyanoacetamide derivative **3** with malononitrile and the same aldehydes (1:1:1 molar ratio) at reflux temperature in the presence of piperidine afforded the 2-pyridone derivatives **7a**–**c**. Spectroscopic data as well as elemental analyses of the obtained products were in complete agreement with the assigned structures **7a**–**c**. In addition, when the cyanoacetamide **3** was reacted with acetylacetone in dioxane in the presence of a catalytic amount of triethylamine, the cyclocondensation reaction occurred and the 2-pyridinone derivatives **10** were smoothly afforded. It can be postulated that the reaction initially proceeds via a nucleophilic attack to form the Michael adduct which in turn cyclized and eliminated two water molecules, affording the final product (Scheme 2).

**Scheme 3.**
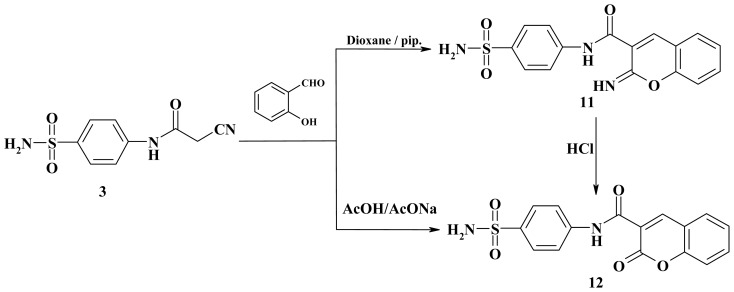
Synthesis of 2-imino-2*H*-chromene **11** and 2-oxo-2*H*-chromene **12**.

Similarly, cyclocondensation of cyanoacetamide **3** with salicyaldehyde in dioxane in the presence of a catalytic amount of piperidine afforded 2-iminochromene **11** in high yield. On the other hand, by interaction of **3** with salicyaldehyde in the presence of AcOH/AcONa, chromenone **12** was obtained in reasonably good yield. The structure of compound **12** was further confirmed through its synthesis upon hydrolysis of **11** with ethanolic HCl. (Scheme 3). The IR spectrum of the reaction product **11** revealed the disappearance of cyano absorption band and showed absorption bands at 1680, 3244 and 3318 cm^−1^ corresponding to carbonyl and two NH functions, respectively. Its ^1^H-NMR spectrum showed two D_2_O-exchangeable signal at δ 9.29 and 13.11 due to two NH protons, in addition to an aromatic multiplet in the region 7.58–7.83. It mass spectrum showed a molecular ion peak at *m*/*z* 343 while ^1^H-NMR spectrum of **12** showed one D_2_O-exchangeable signal at δ 10.88 due to one NH proton.

**Scheme 4.**
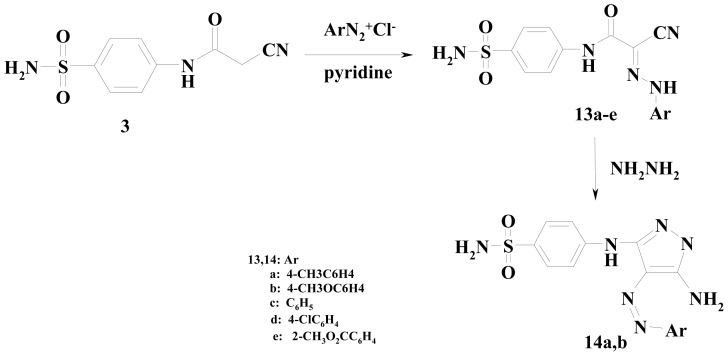
Synthesis of hydrazones **13a**–**e** and aminopyrazole **14a**,**b**.

Next, we studied the reactivity of the active methylene group present in compound **3** towards diazonium salts. Thus, cyanoacetamide **3** coupled with diazonium salts, derived from the appropriate aromatic amines (4-methylaniline, 4-methoxyaniline, aniline, 4-chloroaniline, and methyl anthranilate) in pyridine to afford the respective hydrazones **13a**–**e** (Scheme 4). Analytical and spectral data of the latter reaction products are all consistent with the proposed structures.

Further elucidation of the structure of **13a**,**b** came from the reaction with hydrazine hydrate to furnish the Pyrazole products **14a**,**b**. The structures of compounds **14a**,**b** were confirmed based on elemental analysis and spectral data (see Experimental section).

**Scheme 5.**
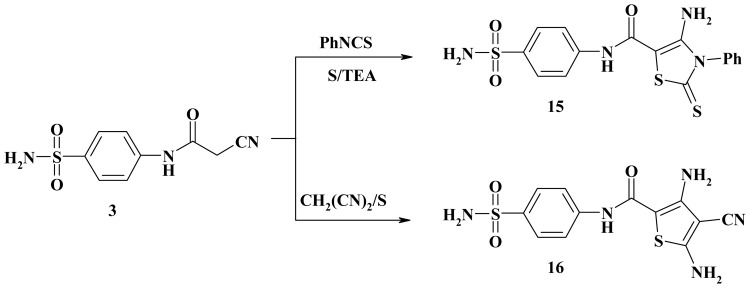
Synthesis of aminothiazole **15** and diaminothiophene **16**.

In view of the growing biological importance of thiazole derivatives, it was considered of interest to synthesize some new derivative of thiazole. Thus, the reaction of compound **3** with phenyl isothiocyanate and elemental sulfur gave the thiazole-2-thione derivative **15**. The reaction of cyanoacetamide **3** with elemental sulfur and malononitrile gave the thiophene derivative **16** (Scheme 5). Analytical and spectral data of the products are in agreement with the proposed structure (see Experimental section).

**Scheme 6.**
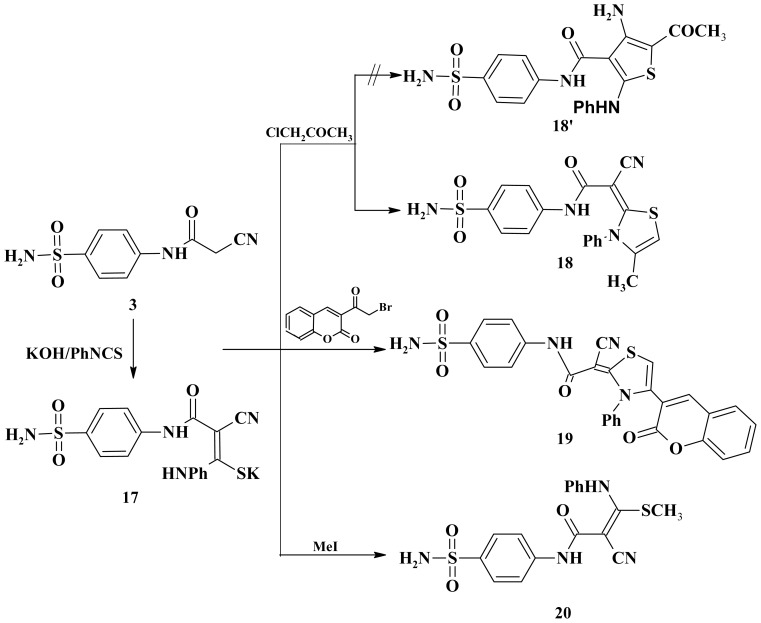
Synthesis of thiazoles **18**, **19** and acrylamide **20**.

The reactivity of cyanoacetamide **3** towards isothiocyanate was investigated. Thus, when **3** was left to react with phenyl isothiocyanate in dimethylformamide, in the presence of potassium hydroxide, at room temperature, the corresponding potassium salt **17** was obtained. Heterocyclization of the intermediate **17** with chloroacetone or 3-(2-bromoacetyl)-2*H*-chromen-2-one furnished in each case, one isolable product (as tested by TLC). Probably the reaction proceeds via nucleophilic displacement of the halogen atom to give an *S*-alkylated intermediate followed by loss of water of the latter intermediate to give thiazole derivatives **18** and **19** as the final products. The structures of the products **18** and **19** as well as the rejection of **18′** were determined from spectroscopic and elemental analytical data. Analytical and spectral data of the product are in agreement with the proposed structure (see Experimental section). Furthermore, the non-isolated potassium salt was methylated by treatment with methyl iodide to afford the novel ketene *N*,*S*-acetal **20** (Scheme 6). The structure of the synthesized product was established on the basis of their elemental analysis and spectral data (see Experimental section).

**Scheme 7.**
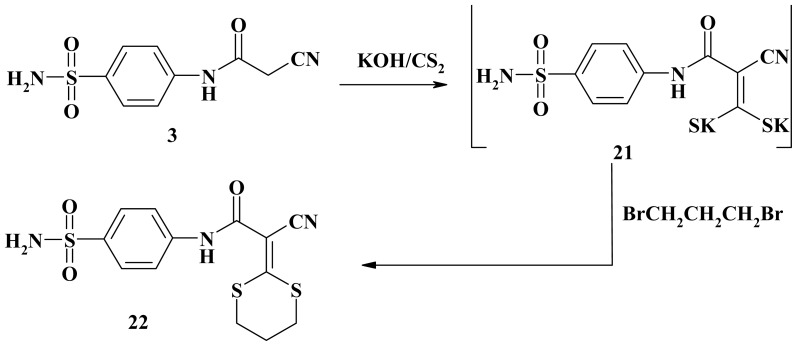
Synthesis of 1,3-dithian-2-ylidene **22**.

Stirring the cyanoacetamide **3** with carbon disulfide in the presence of potassium hydroxide in *N,N*-dimethylformamide followed by cycloalkylation with 1,3-dibromopropane afforded *N*-[4-(amino-sulfonyl)phenyl]-2-cyano-2-(1,3-dithian-2-ylidene)acetamide (**22**) (Scheme 7). The IR spectra of compound **22** showed characteristic bands for NH, CH–aliphatic, C≡N and C=O groups. ^1^H-NMR spectrum of compound **22** showed signals for dithiene moiety at δ 2.18 ppm (m, 2H, *J* = 6.80 Hz, CH_2_), 3.04 (t, 2H, *J* = 6.60 Hz, CH_2_), 3.19 (t, 2H, *J* = 6.60 Hz, CH_2_). Mass spectrum of **22** showed a molecular ion peak at *m*/*z*: 355 with a base peak at *m*/*z*: 183 (100%).

### 2.1. Screening for Antimicrobial Activity

The newly synthesized compounds **5a**, **5c**, **7b**, **7c**, **10**, **11**, **13b**, **14a**, **15**, **16**, **18**, **19** and **22** were evaluated for their *in vitro* antibacterial activity against *Streptococcus pneumoniae* (RCMB-010010) (*SP*) and *Bacillis subtilis* (RCMB-010067) (*BS*) as examples of Gram-positive bacteria and *Pseudomonas aeruginosa* (RCMB-010043) (*PA*) and *Escherichia coli* (RCMB-010052) (*EC*) as examples of Gram-negative bacteria. They were also evaluated for their *in vitro* antifungal activity against *Aspergillus fumigatus* (RCMB-02568) (*AF*), *Syncephalastrum racemosum* (RCMB-05922) (*SR*), *Geotricum candidum* (RCMB-05097) (*GC*) and *Candida albicans* (RCMB-05036) (*CA*) fungal strains. Inhibition zone diameter (IZD) in mm was used as criterion for the antimicrobial activity using the diffusion technique [26–28]. The fungicide Amphotericin B and the bactericides Ampicillin and Gentamicin were used as references to evaluate the potency of the tested compounds under the same conditions. The results are depicted in Table 1. As seen from the data present in Table 1, *Streptococcus pneumoniae* and *Bacillis subtilis* are sensitive to all tested compounds except compounds **11** and **16**; furthermore, *Pseudomonas aeruginosa* is sensitive to compounds **5a**, **7c** and **15**, while *Escherichia coli* is sensitive to **5a**, **7b**, **7c**, **10**, **13b**, **14a**, **15**, **18**, **19** and **22** except compounds **11** and **16**. All tested compounds except compound **16** exhibit antifungal activity against the three tested fungi species *Aspergillus fumigatus*, *Syncephalastrum racemosum* and *Geotricum candidum*. Also the *Candida albicans* strain is sensitive to compounds **5a**, **5c**, **7c**, **10**, **11**. The high activity of **5a**, **7c** and **10** is attributed to the presence of pharmacological active arylidene moiety in compound **5a** and pyridone ring in **7c** and **10**. The inactivity of compound **16** against the tested bacteria and fungi is due to the presence of a thiophene ring.

## 3. Experimental Section

### 3.1. General Experimental Procedures

All melting points were measured on an Electrothermal Gallenkamp apparatus (Weiss-Gallenkamp, London, UK). The infrared spectra were recorded in potassium bromide discs on a Pye Unicam SP3300 and Shimadzo FT IR 8101 PC infrared spectrophotometers (Pye Unicam Ltd., Cambridge, UK and Shimadzu, Tokyo, Japan, respectively). The ^1^H-NMR spectra were recorded on a Varian Mercury VXR-300 spectrometer (300 MHz, Vernon Hills, IL, USA). The mass spectra were recorded on a GCMS-Q1000-EX Shimadzu and GCMS 5988-A HP spectrometers (Kyoto, Japan), the ionizing voltage was 70 eV. Elemental analyses were carried out at the Micro-analytical Center of Cairo University, Giza, Egypt. The biological evaluation of the products was carried out in the Medical Mycology Laboratory of the Regional Center for Mycology and Biotechnology of Al-Azhar University, Cairo, Egypt. The starting material 3-(2-bromoacetyl)-2*H*-chromen-2-one was prepared as previously reported in the literature [29,30].

### 3.2. Synthetic Procedures

#### 3.2.1. 2-Cyano-*N*-(4-sulfamoylphenyl)acetamide (**3**)

A mixture of **1** (3.44 g, 20 mmol) and 3,5-dimetyl-1-cyanoacetyl Pyrazole (**2**) (3.26 g, 20 mmol) in dioxane (20 mL) was refluxed for 3 h. The reaction mixture was poured into crushed ice. The resulting precipitate was filtrated off, dried, and crystallized from DMF/MeOH (1:3) to give **3**; Yield (70%), mp 248 °C (from DMF/MeOH) (lit. mp 230 °C) [31]; IR (KBr) ν_max_: 3337, 3236, 3102 (NH, NH_2_), 2269 (C≡N), 1687 (C=O) cm^−1; 1^H-NMR (DMSO-*d*_6_): δ 3.96 (s, 2H, CH_2_), 7.29 (s, 2H, D_2_O-exchangeable NH_2_), 7.77 (d, 2H, *J* = 9 Hz), 7.80 (d, 2H, *J* = 9 Hz), 10.63 (s, 1H, D_2_O-exchangeable NH). MS *m*/*z* (%): 241 (M^+^ + 2, 0.7), 240 (M^+^ + 1, 5.8), 239 (M^+^, 12.7), 238 (100), 222 (34.9), 172 (25.3), 159 (29.7), 132 (19.1), 108 (47.3), 92 (46.3), 90 (25.1), 75 (15.9). Anal. Calcd for C_9_H_9_N_3_O_3_S (239.25): C, 45.18; H, 3.79; N, 17.56; S, 13.40. Found: C, 45.11; H, 3.70; 65, N, 17.42; S, 13.30%.

#### 3.2.2. *N*-[4-(Aminosulfonyl)phenyl]-3-aryl-2-cyanoacrylamide (**5a**–**c**)

General Procedure: To a solution of cyanoacetanilide **3** (0.239 g, 1 mmol) and the appropriate aromatic aldhyeds (1 mmol) in dioxane (20 mL), was added few drops of piperidine and the reaction mixture was refluxed for 6 h. The solid product so formed was filtered off, washed with EtOH and then recrystallized from proper solvent to give **5a**–**c**.

#### 3.2.3. 2-Cyano-3-phenyl-*N*-(4-sulfamoylphenyl)prop-2-enamide (**5a**)

Yield (45%), mp 322 °C (from dioxane/ethanol); IR (KBr) ν_max_: 3362, 3316, 3262 (NH, NH_2_), 2220 (C≡N), 1680 (C=O) cm^−1; 1^H-NMR (DMSO-*d*_6_): δ 7.29 (s, 2H, D_2_O-exchangeable NH_2_), 7.63 (d, 2H, *J* = 9 Hz), 7.80 (d, 2H, *J* = 9 Hz), 7.81–7.83 (m, 5H, ArH), 8.32 (s, 1H, olefinicH), 10.70 (s, 1H, D_2_O-exchangeable NH). MS *m*/*z* (%): 327 (M^+^, 18.2), 128 (40.4), 104 (10.7), 156 (100), 77 (23.9); Anal. Calcd for C_16_H_13_N_3_O_3_S (327.35): C, 58.70; H, 4.00; N, 12.84; S, 9.80. Found: C, 58.65; H, 3.88; N, 12.72; S, 9.77%.

#### 3.2.4. 2-Cyano-3-(4-methoxyphenyl)-*N*-(4-sulfamoylphenyl)prop-2-enamide (**5b**)

Yield (90%), mp 292 °C (from dioxane/ethanol); IR (KBr) ν_max_: 3312, 3266, 3106 (NH, NH_2_), 2218 (C≡N), 1682 (C=O) cm^−1; 1^H-NMR (DMSO-*d*_6_): δ 3.87 (s, 3H, OCH_3_), 7.19 (d, 2H, *J* = 9 Hz), 7.27 (s, 2H, D_2_O-exchangeable NH_2_), 7.82–7.83 (m, 4H, ArH), 8.04 (d, 2H, *J* = 9 Hz), 8.24 (s, 1H, olefinicH), 10.56 (s, 1H, D_2_O-exchangeable NH); MS *m*/*z* (%): 357 (M^+^, 11.3), 199 (0.3), 187 (12.6), 186 (100), 158 (19.2), 77 (7.6); Anal. Calcd for C17H15N3O4S (357.38): C, 57.13; H, 4.23; N, 11.76; S, 8.97. Found: C, 57.00; H, 4.18; N, 11.55; S, 8.67%.

#### 3.2.5. 2-Cyano-3-(4-chlorophenyl)-*N*-(4-sulfamoylphenyl)prop-2-enamide (**5c**)

Yield (63%), mp 286 °C (from dioxane/ethanol); IR (KBr) ν_max_: 3388, 3329, 3262 (NH, NH_2_), 2218 (C≡N), 1688 (C=O) cm^−1^; ^1^H-NMR (DMSO-*d*_6_): δ 7.37 (s, 2H, D_2_O-exchangeable NH_2_), 7.76 (d, 2H, *J* = 9 Hz), 7.91–8.07 (m, 4H, ArH), 8.10 (d, 2H, *J* = 9 Hz), 8.39 (s, 1H, olefinicH), 10.78 (s, 1H, D_2_O-exchangeable NH); MS *m*/*z* (%): 361 (M^+^, 19.4), 191 (17.3), 190 (100), 162 (27.9), 127 (29.6), 123 (4.6), 111 (5.8), 99 (5.2), 75 (13.8); Anal. Calcd for C_16_H_12_ClN_3_O_3_S (361.80): C, 53.11; H, 3.34; Cl, 9.80; N, 11.61; S, 8.86. Found: C, 53.01; H, 3.29; Cl, 9.72; N, 11.51; S, 8.76%.

#### 3.2.6. Synthesis of Pyridines **7a**–**c**

Method A: A mixture of **5** (10 mmol) and malononitrile (0.66 g, 10 mmol) in ethanol (30 mL) containing piperidine (0.5 mL) was heated under reflux for 3 h. After cooling, the precipitate was filtered off, washed with ethanol and then recrystallized from the proper solvent to give **7a**–**c**.

Method B: Equimolar amounts of **3** (10 mmol) and the appropriate 2-(arylidene)-malononitrile (namely 2-(benzylidene)-malononitrile, 2-(4-methoxybenzylidene)-malononitrile, and 2-(4-chloro benzylidene)-malononitrile) (10 mmol) in ethanol (30 mL) was treated with piperidine (0.5 mL) and the reaction mixture was heated under reflux for 3 h. After cooling, the precipitate was filtered off, washed with ethanol and then recrystallized from the proper solvent to give **7a**–**c**.

Method C: A mixture of **3** (10 mmol), the appropriate aldehyde (namely benzaldehyde, *p*-anisaldehyde, and *p*-chlorobenzaldehyde) (10 mmol), piperidine (10 mmol), and malononitrile (0.66 g, 10 mmol) in ethanol (30 mL) was heated under reflux for 3 h. After cooling, the precipitate was filtered off, washed with ethanol and then recrystallized from the proper solvent to give **7a**–**c**.

#### 3.2.7. 4-(6-Amino-3,5-dicyano-2-oxo-4-phenylpyridin-1(2*H*)-yl)benzenesulfonamide (**7a**)

Yield (54%), mp 316 °C (from dioxane/ethanol); IR (KBr) ν_max_: 3458, 3332, 3212 (NH, NH_2_), 2216 (C≡N), 1672 (C=O) cm^−1^; ^1^H-NMR (DMSO-*d*_6_): δ 7.50 (s, 2H, D_2_O-exchangeable NH_2_), 7.51–7.58 (m, 7H, ArH, NH_2_), 7.60 (d, 2H, *J* = 9 Hz), 8.01 (d, 2H, *J* = 9 Hz); MS *m*/*z* (%): 392 (M^+^ + 1, 10.7), 391 (M^+^, 7.8), 237 (7.8), 155 (9.2), 129 (8.7), 81 (46.5), 69 (100). Anal. Calcd for C_19_H_13_N_5_O_3_S (391.40): C, 58.30; H, 3.35; N, 17.89; S, 8.19. Found: C, 58.20; H, 3.34; N, 17.86; S, 8.15%.

#### 3.2.8. 4-[6-Amino-3,5-dicyano-4-(4-methoxyphenyl)-2-oxopyridin-1(2*H*)-yl]benzenesulfonamide (**7b**)

Yield (56%), mp 350 °C (from dioxane/ethanol); IR (KBr) ν_max_: 3311, 3208, 3078 (NH, NH_2_), 2214 (C≡N), 1659 (C=O) cm^−1^; ^1^H-NMR (DMSO-*d*_6_): δ 3.88 (s, 3H, OCH_3_), 7.12 (s, 2H, D_2_O-exchangeable NH_2_), 7.50–7.52 (m, 6H, ArH, NH_2_), 7.62 (d, 2H, *J* = 9 Hz), 8.00 (d, 2H, *J* = 9 Hz); ^13^C-NMR: δ 55.3, 66.3, 75.4, 87.8, 114.0, 116.5, 120.1, 126.5, 127.7, 129.7, 132.7, 136.8, 145.3, 156.9, 159.5, 161.1; MS *m*/*z* (%): 422 (M^+^ + 1, 15.1), 421 (M^+^, 3.9), 336 (11.2), 265 (12.9), 229 (15.1), 195 (13.6), 185 (34.8), 157 (22.6), 82 (34.4), 55.1 (100). Anal. Calcd for C_20_H_15_N_5_O_4_S (421.43): C, 57.00; H, 3.59; N, 16.62; S, 7.61. Found: C, 56.98; H, 3.53; N, 16.55; S, 7.58%.

#### 3.2.9. 4-[6-Amino-4-(4-chlorophenyl)-3,5-dicyano-2-oxopyridin-1(2*H*)-yl]benzenesulfonamide (**7c**)

Yield (60%), mp > 300 °C (from dioxane/ethanol); IR (KBr) ν_max_: 3348, 3208, 3097 (NH, NH_2_), 2219 (C≡N), 1661 (C=O) cm^−1^; ^1^H-NMR (DMSO-*d*_6_): δ 7.51 (s, 2H, D_2_O-exchangeable NH_2_), 7.56–7.59 (m, 4H, ArH), 7.69 (d, 2H, *J* = 9 Hz), 8.01 (d, 2H, *J* = 9 Hz), 8.10 (s, 2H, D_2_O-exchangeable NH_2_); MS *m*/*z* (%): 427 (M^+^ + 2, 46.3), 426 (M^+^ + 1, 46.0), 425 (M^+^, 100), 397 (38.0), 313 (22.4), 269 (18.4), 156 (25.8), 132 (28.2), 111 (27.3), 106 (22.4), 91 (41.4), 80 (29.1), 77 (55.2). Anal. Calcd for C_19_H_12_ClN_5_O_3_S (425.84): C, 53.59; H, 2.84; Cl, 8.33; N, 16.45; S, 7.53. Found: C, 53.46; H, 2.80; Cl, 8.30; N, 16.42; S, 7.50%.

#### 3.2.10. 4-(3-Cyano-4,6-dimethyl-2-oxopyridin-1(2*H*)-yl)benzenesulfonamide (**10**)

To a mixture of cyanoacetanilide **3** (1.20 g, 5 mmol) and acetylacetone (0.50 g, 1mmol) in dioxane (20 mL), triethylamine (0.5 mL) was added and the reaction mixture was refluxed for 8 h. On cooling, the separated solid was filtered, washed with ethanol and crystallized from DMF to afford the corresponding 4-(3-cyano-4,6-dimethyl-2-oxopyridin-1(2*H*)-yl)benzenesulfonamide (**10**). Yield (64%), mp > 300 °C (from DMF); IR (KBr) ν_max_: 3314, 3174, 3083 (NH, NH_2_), 2223 (C≡N), 1648 (C=O) cm^−1^; ^1^H-NMR (DMSO-*d*_6_): δ 1.98 (s, 3H, CH_3_), 2.40 (s, 3H, CH_3_), 6.49 (s, 1H, pyridineH), 7.52–7.57 (m, 4H, ArH, NH_2_), 7.99 (d, 2H, *J* = 9 Hz, ArH); MS *m*/*z* (%): 303 (M^+^, 51.9), 302 (100), 274 (10.6), 223 (21.5), 171 (0.9), 156 (2.8), 78 (23.6), 50 (20.6). Anal. Calcd for C_14_H_13_N_3_O_3_S (303.33): C, 55.43; H, 4.32; N, 13.85; S, 10.57. Found: C, 55.33; H, 4.28; N, 13.78; S, 10.52%.

#### 3.2.11. 2-Imino-*N*-(4-sulfamoylphenyl)-2*H*-chromene-3-carboxamide (**11**)

A mixture of equimolar amounts of **3** (1.20 g, 5 mmol) and salicyaldehyde (0.61 g, 5 mmol) in 1,4-dioxane (25 mL) containing a catalytic amount of piperidine was heated under reflux for 2 h. The solid product formed was collected by filtration and recrystallized from dioxane/ethanol (3:1) to give **11**. Yield (90%), mp 294 °C (from dioxane/ethanol); IR (KBr) ν_max_: 3318, 3244 (NH, NH_2_), 1680 (C=O) cm^−1^; ^1^H-NMR (DMSO-*d*_6_): δ 7.25 (s, 2H, D_2_O-exchangeable NH_2_), 7.32 (d, 2H, *J* = 9 Hz, ArH), 7.58–7.63 (m, 4H, ArH), 7.83 (d, 2H, *J* = 9 Hz, ArH), 8.58 (s, 1H, CH), 9.29 (s, 1H, D_2_O-exchangeable NH), 13.11 (s, 1H, D_2_O-exchangeable NH); MS *m*/*z* (%): 344 (M^+^ + 1, 4.8), 343 (M^+^, 13.5), 172 (86.5), 156 (62.9), 143 (99.1), 65 (100). Anal. Calcd for C_16_H_13_N_3_O_4_S (343.35): C, 55.97; H, 3.82; N, 12.24; S, 9.34. Found: C, 55.90; H, 3.80; N, 12.20; S, 9.31%.

#### 3.2.12. 2-Oxo-*N*-(4-sulfamoylphenyl)-2*H*-chromene-3-carboxamide (**12**)

Method A: To a solution of **3** (1.20 g, 5 mmol) in acetic acid (30 mL) containing 0.5 g of fused sodium acetate, salicyaldehyde (0.61 g, 5 mmol) was added. The mixture was heated under reflux for 2 h. After cooling, the formed product was collected by filtration and recrystallized from DMF to give **12**.

Method B: The iminochromene derivatives **11** (0.86 g, 2.5 mmol) was dissolved in boiling dioxane (40 mL) and treated with 5 mL HCl. The reaction mixture was heated under reflux for 2 h. Left to cool, the obtained product was filtered off, washed with cold water, and air-dried. Yield (86%), mp > 300 °C (from DMF); IR (KBr) ν_max_: 3362, 3258, 3108 (NH, NH_2_), 1698 (C=O) cm^−1^; ^1^H-NMR (DMSO-*d*_6_): δ 7.30 (s, 2H, D_2_O-exchangeable NH_2_), 7.57 (d, 2H, *J* = 9 Hz, ArH), 7.79–7.89 (m, 4H, ArH), 8.03 (d, 2H, *J* = 9 Hz, ArH), 8.92 (s, 1H, CH), 10.88 (s, 1H, D_2_O-exchangeable NH); ^13^C-NMR: δ 114.9, 118.7, 119.8, 124.3, 126.9, 130.2, 134.6, 139.1, 141.2, 142.1, 153.5, 155.4, 160.2, 161.2; MS *m*/*z* (%): 345 (M^+^ + 1, 3.6), 344 (M^+^, 10.1), 224 (2.8), 173 (100), 118 (5.8), 101 (47.3), 90 (14.6), 80 (14.2), 76 (6.5), 64 (47.0). Anal. Calcd for C_16_H_12_N_2_O_5_S (344.34): C, 55.81; H, 3.51; N, 8.14; S, 9.31. Found: C, 55.76; H, 3.45; N, 8.10; S, 9.29%.

#### 3.2.13. Coupling of *N*-[4-(Aminosulfonyl)phenyl]-2-cyanoacetamide (**3**) with the Appropriate Diazonium Salt of Aromatic Amines

General procedure: To a cold solution of cyanoacetanilide **3** (1.20 g, 5 mmol) in pyridine (20 mL), was added the appropriate diazonium salt of aromatic amine (4-methylaniline or 4-methoxyaniline or aniline or 4-chloroaniline or methyl 2-aminobenzoate) (5 mmol) [prepared according to literature procedures] [32]. The addition was carried out portion wise with stirring at 0–5 °C over a period of 30 min. After complete addition, the reaction mixture was stirred for a further 4 h then kept in an ice chest for 12 h and finally diluted with water. The precipitated solid was collected by filtration, washed with water, dried and finally recrystallized from the proper solvent to afford the corresponding coupling products **13a**–**e**.

#### 3.2.14. 2-Cyano-2-[2-(4-methylphenyl)hydrazinylidene]-*N*-(4-sulfamoylphenyl)ethanamide (**13a**)

Yield (94%), mp 288 °C (from dioxane); IR (KBr) ν_max_: 3325, 3226, 3186 (NH, NH_2_), 2214 (C≡N),1664 (C=O) cm^−1^; ^1^H-NMR (DMSO-*d*_6_): δ 2.30 (s, 3H, CH_3_), 7.20 (s, 2H, D_2_O-exchangeable NH_2_), 7.22–7.64 (m, 4H, ArH), 7.81 (d, 2H, *J* = 9 Hz, ArH), 7.92 (d, 2H, *J* = 9 Hz, ArH), 10.13 (s, 1H, D_2_O-exchangeable NH), 11.93 (s, 1H, D_2_O-exchangeable NH); MS *m*/*z* (%): 358 (M^+^ + 1, 8.7), 357 (M^+^, 45.0), 198 (6.4), 186 (23.8), 172 (87.8), 134 (10.9), 119 (19.9), 106 (47.9), 91 (100), 77 (52.1), 65 (38.3). Anal. Calcd for C_16_H_15_N_5_O_3_S (357.38): C, 53.77; H, 4.23; N, 19.60; S, 8.97. Found: C, 53.71; H, 4.20; N, 19.55; S, 8.87%.

#### 3.2.15. 2-Cyano-2-[2-(4-methoxyphenyl)hydrazinylidene]-*N*-(4-sulfamoylphenyl)ethanamide (**13b**)

Yield (95%), mp 274 °C (from dioxane); IR (KBr) ν_max_: 3336, 3227 (NH, NH_2_), 2212 (C≡N), 1662 (C=O) cm^−1^; ^1^H-NMR (DMSO-*d*_6_): δ 3.77 (s, 3H, OCH_3_), 7.00 (d, 2H, *J* = 9 Hz, ArH), 7.25 (s, 2H, D_2_O-exchangeable NH_2_), 7.69 (d, 2H, *J* = 9 Hz, ArH), 7.81 (d, 2H, *J* = 8.7 Hz, ArH), 7.92 (d, 2H, *J* = 8.7 Hz, ArH), 10.10 (s, 1H, D_2_O-exchangeable NH), 11.95 (s, 1H, D_2_O-exchangeable NH); MS *m*/*z* (%): 374 (M^+^ + 1, 8.7), 373 (M^+^, 24.3), 270 (5.8), 175 (21.4), 172 (39.8 ), 129 ( 22.3), 122 (100), 107 (38.8), 92 (38.8), 77 (47.6). Anal. Calcd for C_16_H_15_N_5_O_4_S (373.38): C, 51.47; H, 4.05; N, 18.76; S, 8.59. Found: C, 51.36; H, 4.00; N, 18.69; S, 8.52%.

#### 3.2.16. 2-Cyano-2-(2-phenylhydrazinylidene)-*N*-(4-sulfamoylphenyl)ethanamide (**13c**)

Yield (92%), mp > 300 °C (from dioxane); IR (KBr) ν_max_: 3367, 3269, 3240 (NH, NH_2_), 2218 (C≡N), 1680 (C=O) cm^−1^; ^1^H-NMR (DMSO-*d*_6_): δ 7.26 (s, 2H, D_2_O-exchangeable NH_2_), 7.38–7.79 (m, 5H, ArH), 7.82 (d, 2H, *J* = 9 Hz, ArH), 7.93 (d, 2H, *J* = 9 Hz, ArH), 10.17 (s, 1H, D_2_O-exchangeable NH), 11.98 (s, 1H, D_2_O-exchangeable NH); MS *m*/*z* (%): 343 (M^+^, 36.8), 206 (21.1), 198 (18.4), 172 (68.4), 145 (50.0), 118 (31.6), 108 (36.8), 91 (84.2), 80 (50.0), 77 (76.3), 60 (100). Anal. Calcd for C_15_H_13_N_5_O_3_S (343.36): C, 52.47; H, 3.82; N, 20.40; S, 9.34. Found: C, C, 52.45; H, 3.78; N, 20.38; S, 9.30%.

#### 3.2.17. 2-[2-(4-Chlorophenyl)hydrazinylidene]-2-cyano-*N*-(4-sulfamoylphenyl)ethanamide **(13d)**

Yield (95%), mp > 300 °C (from dioxane); IR (KBr) ν_max_: 3360, 3232, 3190 (NH, NH_2_), 2216 (C≡N), 1668 (C=O) cm^−1^; ^1^H-NMR (DMSO-*d*_6_): δ 7.26 (s, 2H, D_2_O-exchangeable NH_2_), 7.47 (d, 2H, *J* = 9 Hz), 7.74–7.82 (m, 4H, ArH), 7.92 (d, 2H, *J* = 9 Hz), 10.22 (s, 1H, D_2_O-exchangeable NH), 12.04 (s, 1H, D_2_O-exchangeable NH); MS *m*/*z* (%): 379 (M^+^ + 2, 17.9), 378 (M^+^ + 1, 12.5), 377 (M^+^, 39.3), 296 (26.8), 238 (12.5 ), 206 (41.1), 182 (32.1), 172 (96.4), 156 (51.8), 125 (57.1), 111 (53.6), 106 (30.4), 90 (69.6), 77 (21.4), 64 (100). Anal. Calcd for C_15_H_12_ClN_5_O_3_S (377.80): C, 47.69; H, 3.20; Cl, 9.38; N, 18.54; S, 8.49. Found: C, 47.61; H, 3.15; Cl, 9.27; N, 18.49; S, 8.43%.

#### 3.2.18. Methyl 4-[2-(1-Cyano-2-oxo-2-{(4-sulfamoylphenyl)amino}ethylidene)hydrazinyl]benzoate (**13e**)

Yield (95%), mp > 300 °C (from dioxane); IR (KBr) ν_max_: 3340, 3231, 3148 (NH, NH_2_) 2211 (C≡N), 1687 (C=O) cm^−1^; ^1^H-NMR (DMSO-*d*_6_): δ 3.94 (s, 3H, CH_3_), 7.24 (s, 2H, D_2_O-exchangeable NH_2_), 7.31–7.94 (m, 4H, ArH), 8.03 (d, 2H, *J* = 9 Hz), 8.32 (d, 2H, *J* = 9 Hz), 10.43 (s, 1H, D_2_O-exchangeable NH), 12.49 (s, 1H, D_2_O-exchangeable NH); MS *m*/*z* (%): 402 (M^+^ + 1, 3.0), 401 (M^+^, 12.8), 170 (11.7), 133 (100), 90 (34.1), 92 (20.2), 77 (21.2), 65 (12.7). Anal. Calcd for C_17_H_15_N_5_O_5_S (401.39): C, 50.87; H, 3.77; N, 17.45; S, 7.99. Found: C, 50.81; H, 3.65; N, 17.40; S, 7.92%.

#### 3.2.19. Synthesis of Aminopyrazoles **14a**,**b**

To a solution of the compound **13a**,**b** (5 mmol) in dioxane (20 mL), hydrazine hydrate (80%, 1.0 mL, 5 mmol) was added and the reaction mixture was refluxed for 6 h and allowed to cool. The solid product obtained was filtered, washed with EtOH and dried. Recrystallization from dioxane afforded **14a**,**b**.

#### 3.2.20. 4-({5-Amino-4-[(4-methylphenyl)diazenyl]-1*H*-pyrazol-3-yl}amino)benzenesulfonamide (**14a**)

Yield (40%), mp 240 °C (from dioxane); IR (KBr) ν_max_: 3459, 3367, 3345, 3208 (NH, NH_2_), 2920, 2859 (aliphaticCH) cm^−1^; ^1^H-NMR (DMSO-*d*_6_): δ 2.29 (s, 3H, CH_3_), 5.18 (s, 2H, D_2_O-exchangeable NH_2_), 7.23 (d, 2H, *J* = 9 Hz), 7.42 (d, 2H, *J* = 9 Hz), 7.49 (s, 2H, D_2_O-exchangeable NH_2_), 7.75–7.90 (m, 4H, ArH), 9.86 (s, 1H, D_2_O-exchangeable NH), 13.65 (s, 1H, D_2_O-exchangeable NH); ^13^C-NMR: δ 20.5, 99.4, 117.0, 119.5, 126.4, 129.5, 132.9, 137.9, 141.8, 145.1, 151.5, 165.7; MS *m*/*z* (%): 372 (M^+^ + 1, 14.6), 356 (20.8), 217 ( 31.3 ), 201 (16.7), 156 ( 31.3 ), 126 ( 22.9), 123 (58.3), 107 (50.0), 106 (100), 90 (50.0), 77 (54.2). Anal. Calcd for C_16_H_17_N_7_O_2_S (371.41): C, 51.74; H, 4.61; N, 26.40; S, 8.63. Found: C, 51.70; H, 4.58; N, 26.20; S, 8.59%.

#### 3.2.21. 4-({5-Amino-4-[(4-methoxyphenyl)diazenyl]-1*H*-pyrazol-3-yl}amino)benzenesulfonamide (**14b**)

Yield (72%), mp 200 °C (from dioxane); IR (KBr) ν_max_: 3436, 3351, 3259 (NH, NH_2_), 1658 (C=O) cm^−1^; ^1^H-NMR (DMSO-*d*_6_): δ 3.77 (s, 3H, OCH_3_), 5.03 (s, 2H, D_2_O-exchangeable NH_2_), 7.00 (d, 2H, *J* = 9 Hz), 7.21 (s, 2H, D_2_O-exchangeable NH_2_), 7.49 (d, 2H, *J* = 9 Hz), 7.77–7.81 (m, 4H, ArH), 9.70 (s, 1H, D_2_O-exchangeable NH), 13.20 (s, 1H, D_2_O-exchangeable NH); MS *m*/*z* (%): 389 (M^+^ + 2, 35.4), 388 (M^+^ + 1, 40.2), 387 (M^+^, 38.4), 250 (31.7 ), 216 (40.2), 196 (53.0 ), 171 (45.1), 155 (31.7), 122 (33.5), 108 (34.8), 95 (40.2), 69 (100). Anal. Calcd for C_16_H_17_N_7_O_3_S (387.41): C, 49.60; H, 4.42; N, 25.31; S, 8.28. Found: C, 49.58; H, 4.40; N, 25.30; S, 8.20%.

#### 3.2.22. 4-Amino-3-phenyl-*N*-(4-sulfamoylphenyl)-2-thioxo-2,3-dihydro-1,3-thiazole-5-carboxamide (**15**)

To a solution of cyanoacetamide **3** (1.20 g, 5 mmol) in DMF containing triethylamine (1 mL), elemental sulfur (0.16 g, 5 mmol) and phenyl isothiocyanate (0.68 mL, 5 mmol) were added. The reaction mixture was heated at 60 °C for 2 h with continous stirring and then poured into a beaker containing an ice-water mixture with few drops of HCl. The solid product so formed was filtered off, washed with EtOH and dried. Recrystallization from dioxane afforded compound **15**. Yield (60%), mp > 300 °C (from DMF); IR (KBr) ν_max_: 3397, 3210 (NH, NH_2_), 1671 (C=O), 1335, 1217 (C=S) cm^−1^; ^1^H-NMR (DMSO-*d*_6_): δ 3.42 (s, 2H, D_2_O-exchangeable NH_2_), 7.19 (s, 2H, D_2_O-exchangeable NH_2_), 7.50 (d, 2H, *J* = 9 Hz), 7.55–7.88 (m, 5H, ArH), 7.96 (d, 2H, *J* = 9 Hz), 8.45 (s, 1H, D_2_O-exchangeable NH); MS *m*/*z* (%) 406 (M^+^, 0.9), 402 (5.7), 172 (14.3), 156 (8.6), 129 (11.4), 114 (11.4), 109 (17.1), 82 (32.9), 76 (44.3), 63 (100). Anal. Calcd for C_16_H_14_N_4_O_3_S_3_ (406.50): C, 47.27; H, 3.47; N, 13.78; S, 23.66. Found: C, 47.21; H, 3.44; N, 13.71; S, 23.59%.

#### 3.2.23. 3,5-Diamino-4-cyano-*N*-(4-sulfamoylphenyl)thiophene-2-carboxamide (**16**)

To a solution of compound **3** (1.20 g, 5 mmol) in dioxane (25 mL) containing triethylamine (1.00 mL), malononitrile (0.33 g, 5 mmol) was added followed by the addition of an equimolar amount of elemental sulfur (0.16 g, 5 mmol). The reaction mixture was heated under reflux for 5 h, then cooled and neutralized by pouring onto ice/water mixture containing few drops of hydrochloric acid. The solid product formed was collected by filtration and crystallized from dioxane. Yield (86%), mp > 300 °C (from dioxane); IR (KBr) ν_max_: 3743, 3316 (NH, NH_2_), 2209 (C≡N), 1635 (C=O) cm^−1^; ^1^H-NMR (DMSO-*d*_6_): δ 3.45 (s, 4H, D_2_O-exchangeable 2NH_2_), 7.26 (s, 2H, D_2_O-exchangeable NH_2_), 7.50–7.92 (m, 4H, ArH), 11.36 (s, 1H, D_2_O-exchangeable NH); MS *m*/*z* (%): 338 (M^+^ + 1, 44.9), 337 (M^+^, 47.2), 295 (47.2), 278 (45.7), 181 (49.6), 156 (53.5), 150 (51.9), 122 (51.2), 105 (64.6), 80 (48.8), 58 (100.0). Anal. Calcd for C_12_H_11_N_5_O_3_S (337.37): C, 42.72; H, 3.29; N, 20.76; S, 19.01. Found: C, 42.68; H, 3.21; N, 20.66; S, 18.96%.

#### 3.2.24. Synthesis of **18**, **19** and **20**

Compound **3** (1.20 g, 5 mmol) was added to a stirred solution of potassium hydroxide (0.26 g, 5 mmol) in DMF (20 mL). After stirring for 30 min, phenyl isothiocyanate (0.68 g, 5 mmol) was added to the resulting mixture. Stirring was continued for 6 h, and then chloroacetone or 3-(2-bromoacetyl)- 2*H*-chromen-2-one or methyl iodide, (5 mmol) was added portion wise over a period of 30 min. After the addition was complete, the reaction mixture was stirred for an additional 12 h, during which the reactant dissolved and a yellow product precipitated. The solid product was filtered off, washed with EtOH and dried. Recrystallization from proper solvent afforded **18**, **19** and **20**.

#### 3.2.25. *N*-[4-(Aminosulfonyl)phenyl]-2-cyano-2-(4-methyl-3-phenyl-1,3-thiazol-2(3*H*)-ylidene)acet- amide (**18**)

Yield (95%), mp 272 °C (from Dioxane) (lit. mp 260–262 °C) [31]; IR (KBr) ν_max_: 3294, 3196, 3107 (NH, NH_2_), 2178 (C≡N), 1605 (C=O) cm^−1^; ^1^H-NMR (DMSO-*d*_6_): δ 1.86 (s, 3H, CH_3_), 6.98 (s, 1H, thiazole-CH), 7.17 (s, 2H, D_2_O-exchangeable NH_2_), 7.49 (d, 2H, *J* = 9 Hz), 7.57 (d, 2H, *J* = 9 Hz), 7.60–7.70 (m, 5H, ArH), 9.02 (s, 1H, D_2_O-exchangeable NH); ^13^C-NMR (DMSO-*d*_6_): δ 14.2, 66.3, 106.9, 115.9, 119.3, 126.2, 128.9, 129.6, 130.6, 136.6, 137.5, 138.3, 142.4, 165.1, 166.5; MS *m*/*z* (%): 412 (M^+^, 10.1), 332 (2.1), 241 (100), 214 (35.3), 198 (19.2), 172 (6.9), 118 (19.9), 90 (38.3), 77 (43.2). Anal. Calcd for C_19_H_16_N_4_O_3_S_2_ (412.48): C, 55.32; H, 3.91; N, 13.58; S, 15.55. Found: C, 55.30; H, 3.88; N, 13.51; S, 15.50%.

#### 3.2.26. 2-Cyano-2-[4-(2-oxo-2*H*-chromen-4-yl]-3-phenyl-1,3-thiazol-2(3*H*)-ylidene)-*N*-(4-sulfamoyl- phenyl)ethanamide (**19**)

Yield (55%), mp 280 °C (from Dioxane); IR (KBr) ν_max_: 3367, 3310 and 3235 (NH, NH_2_), 3060 (aromatic CH), 1713 (C=O), 1635 (C=O) cm^−1^; ^1^H-NMR (DMSO-*d*_6_): δ 1.01 (s, 3H, *J* = 7.2 Hz, CH_3_), 4.04 (q, 2H, *J* = 7.2 Hz, CH_2_), 7.08–7.13 (m, 5H, ArH), 7.21 (d, 2H, *J* = 9 Hz), 7.39–7.44 (m, 7H, ArH and NH_2_), 7.63 (d, 2H, *J* = 9 Hz), 9.54 (s, 1H, D_2_O-exchangeable NH), 9.73 (s, 1H, D_2_O-exchangeable NH); ^13^C-NMR: δ 70.9, 95.3, 115.4, 115.8, 118.0, 119.6, 120.9, 124.8, 125.8, 126.3, 128.4, 128.8, 129.3, 129.5, 132.7, 137.0, 138.0, 142.1, 143.0, 153.2, 158.4, 164.7, 171.3; MS *m*/*z* (%): 521 (M^+^, 26.3), 322 (15.8), 218 (100.0), 199 (15.8), 77 (86.0). Anal. Calcd for C_27_H_18_N_4_O_5_S_2_ (542.58): C, 59.77; H, 3.34; N, 10.33; S, 11.82. Found: C, 59.75; H, 3.30; N, 10.31; S, 11.79%.

#### 3.2.27. 2-Cyano-3-(methylsulfanyl)-3-(phenylamino)-*N*-(4-sulfamoylphenyl)prop-2-enamide (**20**)

Yield (95%), mp 204 °C (from EtOH) (lit. mp 220–222 °C) [33]; IR (KBr) ν_max_: 3410, 3339, 3243, 3119 (NH, NH_2_), 2194 (C≡N), 1628 (C=O) cm^−1^; ^1^H-NMR (DMSO-*d*_6_): δ 2.49 (s, 3H, CH_3_), 7.18 (s, 2H, D_2_O-exchangeable NH_2_), 7.31–7.41 (m, 5H, ArH), 7.67 (d, 2H, *J* = 9 Hz), 7.79 (d, 2H, *J* = 9 Hz), 9.65 (s, 1H, D_2_O-exchangeable NH), 9.91 (s, 1H, D_2_O-exchangeable NH); MS *m*/*z* (%): 389 (M^+^ + 1, 63.0), 388 (M^+^, 10.9), 341 (58.7), 294 (100), 249 (56.5), 223 (67.4), 208 (68.5), 197 (58.7), 185 (25.0), 182 (59.8), 140 (76.1), 92 (17.4). Anal. Calcd for C_17_H_16_N_4_O_3_S_2_ (388.46): C, 52.56; H, 4.15; N, 14.42; S, 16.51. Found: C, 52.50; H, 4.12; N, 14.45; S, 16.48%.

#### 3.2.28. 2-Cyano-2-(1,3-dithian-2-ylidene)-*N*-(4-sulfamoylphenyl)acetamide (**22**)

To a stirred suspension of finely powdered potassium hydroxide (0.26 g, 5 mmole) in dry DMF (20 mL) cyanoacetamide **3** (1.20 g, 5 mmole) was added, the resulted mixture was cooled at 10 °C in an ice bath, then carbon disulfide (5 mmol) was added slowly over the course of 10 min. After addition was complete, stirring of the reaction mixture was continued for additional 2 h. Then dibromopropane (5 mmol) was added to the mixture while cooling (~15 °C) and stirring for 1 h. The mixture was then poured into crushed ice and the resulting precipitate was filtrated off, dried and crystallized from the proper solvent to give **22**. Yield (95%), mp 278 °C (from Dioxane); IR (KBr) ν_max_: 3314, 3241, 3107 (NH, NH_2_), 2209 (C≡N), 1653 (C=O) cm^−1^; ^1^H-NMR (DMSO-*d*_6_): δ 2.18 (m, 2H, *J* = 6.80 Hz, CH_2_), 3.04 (t, 2H, *J* = 6.6 Hz, CH_2_), 3.19 (t, 2H, *J* = 6.6 Hz, CH_2_), 7.25 (s, 2H, D_2_O-exchangeable NH_2_), 7.75 (m, 4H, ArH), 10.29 (s, 1H, D_2_O-exchangeable NH); ^13^C-NMR: δ 22.5, 29.2, 98.5, 115.6, 119.8, 126.5, 139.0, 141.3, 159.8, 177.3; MS *m*/*z* (%): 356 (M^+^ + 1, 2.9), 355 (M^+^, 14.8), 237 (0.2), 183 (100), 129 (2.3), 118 (1.2), 110 (27.6), 80 (18.1). Anal. Calcd for C_13_H_13_N_3_O_3_S_3_ (355.45): C, 43.93; H, 3.69; N, 11.82; S, 27.06. Found: C, 43.85; H, 3.57; N, 11.78; S, 27.00%.

### 3.3. Antimicrobial Evaluation

The antibacterial and antifungal activity assays were carried out in the Medical Mycology Laboratory of the Regional Center for Mycology and Biotechnology of Al-Azhar University, Cairo, Egypt using the diffusion plate method [26–28] as follows: a bottomless cylinder containing a measured quantity (1 mL, 5 mg/mL) of the sample is placed on a plate (9 cm diameter) containing a solid bacterial medium (nutrient agar broth) or fungal medium, which has been heavily seeded with a spore suspension of the test organism. After incubation (24 h for bacteria and 5 days for fungi), the diameter of the clear zone of inhibition surrounding the sample is taken as measure of the inhibitory power of the sample against the particular test organism. The solvent used was DMSO and the concentration of the sample used is 100 μg/mL. The results of antimicrobial activity are summarized in Table 1.

## 4. Conclusions

In conclusion, the reactivity of *N*-[4-(aminosulfonyl)phenyl]-2-cyanoacetamide (**3**) was investigated as a versatile and readily accessible building block for the synthesis of new heterocycles incorporating a sulfamoyl moiety of biological and pharmaceutical importance.

## Figures and Tables

**Table 1.** Antibacterial and antifungal activities of the synthesized compounds (**5a**, **5c**, **7b**, **7c**, **10**, **11**, **13b**, **14a**, **15**, **16**, **18**, **19** and **22**).

Comp.	Inhibition zone diameter (cm)							

	Gram (+)		Gram (−)		Fungi			

Standard values	*(SP)*	*(BS)*	*(PA)*	*(EC)*	*(AF)*	*(SR)*	*(GC)*	*(CA)*

	23.8 ± 0.2	32.4 ± 0.3	17.3 ± 0.1	19.9 ± 0.3	23.7 ± 0.2	19.7 ± 0.2	28.7 ± 0.2	25.4 ± 0.1
**5a**	18.9 ± 0.44	21.7 ± 0.25	11.6 ± 0.19	15.4 ± 0.39	20.2 ± 0.55	16.3 ± 0.25	22.4 ± 0.58	19.6 ± 0.33
**5c**	16.3 ± 0.55	18.3 ± 0.25	NA	NA	17.3 ± 0.44	12.6 ± 0.25	19.0 ± 0.58	16.9 ± 0.25
**7b**	16.9 ± 0.58	18.2 ± 0.44	NA	11.9 ± 0.63	15.7 ± 0.33	13.8 ± 0.25	18.3 ± 0.34	NA
**7c**	18.3 ± 0.25	22.6 ± 0.44	13.1 ± 0.32	20.3 ± 0.09	20.6 ± 0.58	16.7 ± 0.33	22.4 ± 0.36	17.6 ± 0.58
**10**	16.7 ± 0.36	19.2 ± 0.27	NA	13.6 ± 0.36	16.8 ± 0.39	13.4 ± 0.58	19.6 ± 0.19	15.9 ± 0.44
**11**	NA	NA	NA	NA	15.7 ± 0.36	11.2 ± 0.33	17.3 ± 0.44	13.3 ± 0.36
**13b**	12.3 ± 0.58	12.7 ± 0.37	NA	8.5 ± 0.37	17.6 ± 0.58	15.4 ± 0.25	12.6 ± 0.38	NA
**14a**	17.5 ± 0.44	19.8 ± 0.63	NA	18.9 ± 0.25	15.3 ± 0.55	13.4 ± 0.35	11.5 ± 0.58	NA
**15**	15.0 ± 0.43	17.4 ± 0.53	12.3 ± 0.25	17.8 ± 0.03	11.3 ± 0.34	12.1 ± 0.25	15.3 ± 0.38	NA
**16**	NA	NA	NA	NA	NA	NA	NA	NA
**18**	16.9 ± 0.58	18.2 ± 0.44	NA	11.9 ± 0.63	16.2 ± 0.36	15.0 ± 0.44	17.6 ± 0.58	NA
**19**	12.9 ± 0.63	13.2 ± 0.58	NA	10.8 ± 0.44	18.7 ± 0.36	16.9 ± 0.27	13.4 ± 0.65	NA
**22**	12.3 ± 0.58	12.7 ± 0.37	NA	10.8 ± 0.44	17.6 ± 0.58	15.4 ± 0.25	12.6 ± 0.38	NA

Data are expressed in the form of mean ± SD. Mean zone of inhibition in mm ± standard deviation beyond well diameter; (6 mm) produced on a range of environmental and clinically pathogenic microorganism using (5 mg/mL) concentration of tested sample (100 μL was tested).
